# Pre-ART retention in care and prevalence of tuberculosis among HIV-infected children at a district hospital in southern Ethiopia

**DOI:** 10.1186/1471-2431-14-250

**Published:** 2014-10-04

**Authors:** Emil Westerlund, Degu Jerene, Zewdie Mulissa, Inger Hallström, Bernt Lindtjørn

**Affiliations:** Faculty of Medicine, Lund University, Lund, Sweden; Department of Preventive Medicine, Addis Ababa University, Addis Ababa, Ethiopia; Management Sciences for Health-HEAL TB Project, Addis Ababa, Ethiopia; Columbia University- International center for AIDS care and treatment program, Addis Ababa office, Addis Ababa, Ethiopia; Centre for International Health, University of Bergen, Bergen, Norway

**Keywords:** HIV, TB, Ethiopia, Children, ART, Arba Minch, Resource-limited, WHO

## Abstract

**Background:**

The Ethiopian epidemic is currently on the wane. However, the situation for infected children is in some ways lagging behind due to low treatment coverage and deficient prevention of mother-to-child transmission. Too few studies have examined HIV infected children presenting to care in low-income countries in general. Considering the presence of local variations in the nature of the epidemic a study in Ethiopia could be of special value for the continuing fight against HIV. The aim of this study is to describe the main characteristics of children with HIV presenting to care at a district hospital in a resource-limited area in southern Ethiopia. The aim was also to analyse factors affecting pre-ART loss to follow-up, time to ART-initiation and disease stage upon presentation.

**Methods:**

This was a prospective cohort study. The data analysed were collected in 2009 for the period January 2003 through December 2008 at Arba Minch Hospital and additional data on the ART-need in the region were obtained from official reports.

**Results:**

The pre-ART loss to follow-up rate was 29.7%. Older children (10–14 years) presented in a later stage of their disease than younger children (76.9% vs. 45.0% in 0–4 year olds, chi-square test, χ2 = 8.8, P = 0.01). Older girls presented later than boys (100.0% vs. 57.1%, Fisher’s exact test, P = 0.02). Children aged 0–4 years were more likely to be lost to follow-up (40.0 vs. 21.8%, chi-square test, χ2 = 5.4, P = 0.02) and had a longer time to initiate ART (Cox regression analysis, HR: 0.50, 95% CI: 0.25-0.97, P = 0.04, controlling for sex, place of residence, enrolment phase and WHO clinical stage upon presentation). Neither sex was overrepresented in the sample. Tuberculosis prevalence upon presentation and previous history of tubercolosis were 14.5% and 8% respectively.

**Conclusions:**

The loss to follow-up is alarmingly high and children present too late. Further research is needed to explore specific causes and possible solutions.

**Electronic supplementary material:**

The online version of this article (doi:10.1186/1471-2431-14-250) contains supplementary material, which is available to authorized users.

## Background

Recent global reports suggest considerably improved access to antiretroviral therapy (ART) in low and middle-income countries including in Sub-Saharan Africa. According to the 2013 global report, 9.7 million patients were receiving life-saving ART by the end of 2012 [1]. This is a remarkable acheivement as compared to a decade earlier when less than half a million patients were on ART in low and middle-income countries [2]. However, these gains in access are being challenged by emerging set of problems, one of which being retaining patients in care. A recent systematic review revealed problems with patient retention both before and after initiating ART [3]. This challenge and its associated factors are more clearly delineated in adult patient populations than in children.

During 2011, about 24 000 new infections were reported to have occurred and there were around 790 000 people infected with HIV living in Ethiopia, which is 1.5% of the entire population. The latter prevalence is projected to decline as well, along with mortality and incidence [4]. These encouraging results are believed to be the effect of concerted local and global actions including free delivery of HIV services at point of care, service decentralization, and task shifting [5, 6]. The challenge of poor linkage to and retention in care has been well recognized in the Ethiopian HIV program. Published studies from Arba Minch and Gondar hospitals, for example, suggest high rates of pre-ART patient loss among adult patients living with HIV [6, 7]. As elsewhere, there is limited information on the challenges of pre-ART patient retention among children living with HIV in Ethiopia [2].

Despite the success, many challenges still prevail [2, 8]. There are problems with low condom use, violence against women and stigma leading to loss of job and income. One in five Ethiopians with HIV experience suicidal feelings. Another major problem is the low coverage of antiretroviral regimens to prevent mother-to-child transmission and rates of infant testing and prophylaxis remain very low [2, 9].

Some of the most worrying statistics on HIV in Ethiopia concern the health of children. The deficient prevention of mother-to-child transmission in Ethiopia is central to the problem of HIV among children. Ethiopian studies have shown, similarly to findings from other low-income countries and international reports, that a majority of children with HIV in the country have acquired their infection vertically [9–11]. Apart from this, a big problem is the low ART coverage for children. Although the data are currently under review and may reveal an improvement, reports from 2010 announce that only 14–38% of the ART-need among children is met [9].

For those children who do present to care, further problems ensue. Timely initiation of treatment and retention in care are both crucial for patient outcome [12, 13]. Late presentation to care among children have been identified as a problem in both high-income and low-income countries [11, 14]. This implies that children in low-income countries are put in a particularly vulrerable situation as late presentation has been shown to be also a general problem in Sub-Saharan Africa [6, 15]. A review from 2009 has found the reasons for this to be mainly population-based barriers to access such as lack of information, stigma and perceived high cost of treatment. However, the reviewer stresses that ‘there is a paucity of studies on access barriers to ART for HIV-positive children’ [16]. Furthermore, not all studies show that children present late. For instance, a study from India showed that children below 14 years of age actually were associated with a lower risk of late presentation [17]. This further underscores the importance of more local research. Extending the knowledge on HIV-infected children presenting to care in Ethiopia is critical for not letting children lag behind in the process to overcome the epidemic.

In this report, we present data from one of the longest-followed cohorts of paediatric HIV patients on TB prevalence and the magnitude and predictors of retention in care. The aim of this study was to describe the main characteristics and to analyze the predictors of pre-ART loss to follow up among children with HIV presenting to care at a district hospital in a resource-limited area in southern Ethiopia.

## Methods

### Participants

The data were collected at Arba Minch Hospital in southern Ethiopia. The hospital is a general public hospital located in the city of Arba Minch, in the Southern Nations, Nationalities, and People’s Region (SNNPR), around 500 kilometres south of Addis Ababa, Ethiopia’s capital. Arba Minch Hospital was the first hospital to introduce antiretroviral treatment in SNNPR in 2003—at a time when there were only few such centres in Ethiopia. The hospital serves a population of over 1.5 million in the Gamo Goffa zone of SNNPR. We established the HIV cohort database in 2003 and maintained it through this date. Since this is the longest-followed cohort in Ethiopia and resource constraint did not allow us to establish more of such centres, we opted to continue with analysis and learning from our existing cohort. This study did not entail any active data collection. We used de-identified data from the existing database and restricted our analysis to the paediatric age group as most of our earlier analyses did not involve this age group. All children with HIV presenting to care at Arba Minch Hospital from January 2003 through December 2008 were included in this study. The inclusion criteria were to be under 15 years old and to have an HIV infection. Children with a previous history of ART were excluded.

### Procedure of therapy and data collection

The data for this study were collected along with data for adult patients at the same hospital. The findings on those data have been published elsewhere and the method described below is in part described in that study as well [6]. A trained health care worker did the initial evaluation and subsequent follow-up of the patients. During the initial years of the study this evaluation was made using only clinical and total lymphocyte count (TLC) criteria. From mid-2006 and onward CD4 testing was also available. The patients started ART according to the national guidelines issued by the Ethiopian Ministry of Health (MOH), which issued updated versions of the guidelines during the course of this study.

The first Ethiopian ART guidelines were published in 2003 and the paediatric treatment guidelines were included as a chapter in the adult guidelines. The first paediatric ART guidelines were published in 2008 and the recommendations did not change since then. Accordingly, treatment is recommended for all infants with confirmed HIV infection. Treatment is recommended for older children with stage III or IV diseases irrespectively of CD4 count/percentage. For those with stage I or II disease a table is used as a guide outlining different CD4 count/percentage thresholds for different ages (12–35 months: < 750 cells/mm^3^ or < 20%, 36–59 months: < 350 cells/mm^3^ or < 20%, 5 years or older - same as adults: < 200 cells/mm^3^ or < 15%). These thresholds did slightly change during the course of the study. In the 2003 guidelines the 15% threshold extended down to children of 18 months and the adult thresholds were applied to children 8 years or older. The guidelines also specify how to assess HIV infection and disease stage clinically if proper laboratory tests are unavailable [18–20].

Two data clerks recorded patient information both on paper and electronically. With a data abstraction form as a guide they recorded variables directly into an SPSS file. These variables include date of HIV-testing, date of pre-ART enrolment, WHO clinical stage, total lymphocyte count (TLC), CD4 count, haemoglobin level (HGB), history of TB (as reported by patient or caregiver), current TB (diagnosed within one month before or after presentation), sex, age, place of residence (rural or urban), pre-ART outcome and date of ART-initiation (if initiated).

During most of the time of the study, there was no strict guideline for pre-ART follow-up schedule neither for adults nor for children. Patients at the hospital were told to return after 3–6 months depending on their clinical condition. The 2008 paediatric guidelines formalised the schedule to recommend follow-up every 1–3 months depending on age and clinical condition (or more frequently than monthly if clinically indicated) [20]. However, there was no recording and reporting mechanism for the pre-ART visits and definitions for pre-ART outcomes such as loss to follow-up was not formalized. The Ethiopian Ministry of Health has recently finalized a nation-wide assessment of the status of pre-ART patient care. It is expected to lead to development of a comprehensive national framework for pre-ART care. In the mean time, we continued to use our own operational definition for pre-ART care. The pre-ART outcome was defined as: (a) ‘still under pre-ART care’ – if the patient was registered with the ART clinic of the hospital, had regular follow-up with the clinic and was not having follow up at another health facility; (b) ‘lost to follow-up’ – if patient did not have a follow-up visit at least 30 days after the last date of the most recent clinic appointment; (c) ‘put on ART’ – if patient was started on ART in the hospital clinic; (d) ‘died before starting ART’ – if patient was known to be dead as reported by treating clinicians or community health agents; and (e) ‘transferred out’ – if patient moved to another health facility with confirmed written documentation of transfer out.

For those patients that were put on ART, patients were defined as lost to follow-up if they had not attended the hospital within 30 days following the time for their clinical appointment. For patients lost to follow-up an extended follow-up was conducted in 2009 and involved a home visit or phone call using community health agents. Patient status after extended follow-up was defined as (a) ‘died’ – if a family member, neighbour or community leader reported death of the patient; (b) ‘under follow up at another health facility’ – if the patient was on treatment at any health facility in the region as reported by family, neighbours or community leaders; (c) ‘stopped treatment but alive’ – if the patient had not taken antiretroviral drugs (ARVs) for over a month and the patient was alive and did not get ART elsewhere; (d) ‘on traditional treatment’ – if the patient reported that he or she used traditional medicines or treatment instead of ART and (e) ‘left the region’ – if patient left the region as reported by family, neighbours or community leaders. If no information was available about the patient, this was defined as (f) ‘unknown’ (‘true loss’).

Patient data were updated at each visit. The database was updated on a quarterly basis 2003–2006 and yearly the last two years. In 2009, we undertook a more thorough cohort updating that involved home visits to determine the status of each patient declared to be lost as described above. The recorded data were updated, amended and cross-checked with paper records at the hospital in order to affirm their quality. In addition to the patient data, data on ART-need among girls and boys in SNNPR were obtained from an official report for statistical comparison with data on the participants of the study [4].

### Ethics

The prospective cohort follow up system at Arba Minch hospital was established with the approaval of the the National Research Ethics Review Committee in Ethiopia. All patients were given standard care at the hospital, as prescribed in the national guidelines [18, 19].

This particular analysis was done based on a separate protocol specifically designed to look into long-term treatment outcomes including pre-ART outcomes for which separate local approval was sought and granted. Since we used de-identified data for this analysis, obtaining patient consent was not feasible but permission was obtained from the hospital administration.

### Statistical methods

SPSS was used for the analyses presented in this study. The data was entered into SPSS version 16 and later transferred to SPSS version 21. All data used for describing cohort profile and baseline characteristics were obtained from the SPSS file.

Data were grouped into three cohorts based on date of enrolment to pre-ART care and the chronology of Ethiopia’s ART scale-up [5]. The three cohorts were decided to be (i) those enrolled January 2003-August 2006 (Early cohort), (ii) those enrolled September 2006-August 2007 (Rapid scale-up cohort) and (iii) those enrolled between September 2007-December 2008 (Recent cohort). For each cohort the proportion of patients presenting in the different WHO clinical stages of HIV/AIDS was compared. For the sake of clarity, a comparison was also made with the WHO clinical stage dichotomized into less advanced (stages I & II combined) and advanced (stages III and IV). In regard to the small sample size these distributions were only described for each cohort separately and no trend analyses were performed.

Logistic regression including the dichotomized WHO stage variable was used to identify potential risk factors for being in an advanced stage upon presentation. Because of the small sample size only four variables were screened: age, sex, place of residence and cohort. These variables were chosen on bases of biological and social plausibility and on the findings from the adult cohort [6]. A similar logistic regression was used to determine risk factors for being lost to pre-ART follow-up and the same variables were chosen for this analysis. Individual chi-square tests were performed to further analyse factors found to affect pre-ART loss and late presentation. For one analysis where the criteria for performing a chi-square test were not deemed to be met, Fischer’s exact test was done instead.

Student’s T test was used to determine whether the distribution between boys and girls presenting to care was significantly different from the distribution among HIV-infected children in general. Estimates for these numbers were obtained from official reports on the region, issued by the Ethiopian Ministry of Health [4].

Time to ART-initiation for different age groups was estimated using Cox regression, controlling for sex, place of residence, enrolment phase and WHO clinical stage upon presentation. Statistical significance was defined as P < 0.05.

The research adhered to strengthening the reporting of observational studies in epidemiology (STROBE) guidelines [21] (See Additional file 1 for more details).

## Results

### Cohort profile

**Figure 1:**
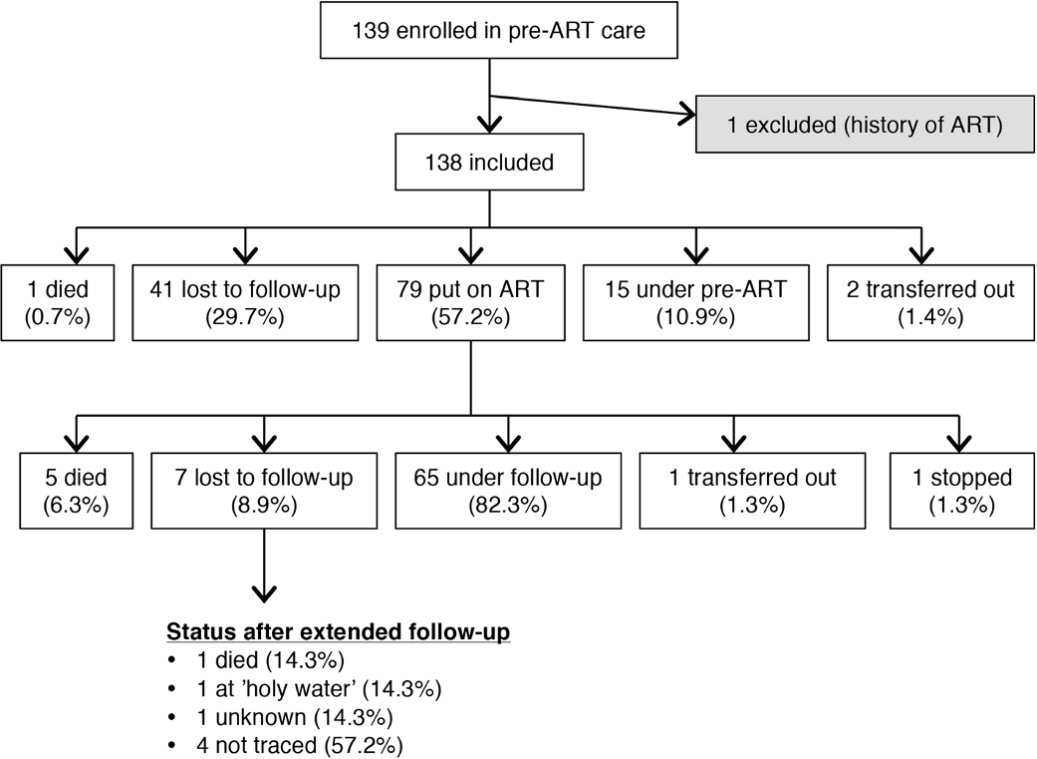
**Cohort profile.** Cohort profile of children treated at Arbaminch Hospital during the period 2003–2008, Arba Minch, Ethiopia.

Out of the 139 children who initiated pre-ART care from January 2003 through December 2008 all but one were enrolled in the study. The child who was not enrolled had a history of previous ART and thus failed to meet the inclusion criteria. Out of the 138 children included, 79 (57.2%) were put on ART, 15 (10.9%) were still under pre-ART care at the time of follow up, two (1.4%) had been transferred out of the hospital and one child (0.7%) had died. The remaining 41 children (29.7%) were lost to follow-up (Figure 1).

Of the 79 children who were put on ART, a majority was still enrolled in treatment at the time of follow-up, namely 65 children (82.3%). Five children (6.3%) had died, one (1.3%) had been transferred out and one had stopped treatment. The remaining 7 children (8.9%) were lost to follow-up. Three of these children were living in urban addresses and were traced for an extended follow-up. One had died and another was alive, receiving traditional treatment at ‘holy water’. The outcome of the last child remains unknown.

The 138 patients enrolled in the study contributed 175.9 person years of observation (PYO). The median time to pre-ART outcome was 1.1 months (IQR: 0.2-6.1) and the median time from ART initiation to ART outcome (for the 79 patients put on ART) was 23.3 months (IQR: 5.1-30.1).

### Characteristics of the children

The characteristics of the 138 children upon enrolment to pre-ART care are shown in Table 1. Their median age was 5 years (IQR: 3–8); 60 children (43.5%) were 0–4 years old, 52 children (37.7%) were 5–9 years old and 26 children (18.8%) were 10–14 years old. As for distribution between sexes, there were 79 boys (57.0%) in the entire sample. A vast majority of 121 children (87.7%) were urban residents and the remaining 17 (12.3%) had rural addresses. A previous history of TB was found for 11 children (8.0%) while 20 children (14.5%) had a TB infection upon presentation. CD4 count was recorded for 97 patients and the mean value was 529 cells/mm^3^.

**Table 1:** **Presenting characteristics of children, Arba Minch Hospital, Ethiopia**

Characteristic		Number (%)
Age	0-4 years	60 (43.4)
	5-9 years	52 (37.7)
	10-14 years	26 (18.8)
Sex	Female	59 (42.8)
	Male	79 (57.2)
Place of residence	Urban	121 (57.2)
	Rural	17 (12.3)
WHO clinical stage	Stage I	31 (22.5)
	Stage II	37 (26.8)
	Stage III	59 (42.8)
	Stage IV	11 (8.0)
Past history of TB	Yes	11 (8.0)
	No	127 (92.0)
TB upon presentation	Yes	20 (14.5)
	No	118 (85.5)
**Characteristic**		**Central tendency (variation)**
CD4 count^*^	Mean	529 cells/mm^3^
Hgb^**^	Mean	10.7 g/dl
Time to ART^***^	Median (IQR)	18 days (6–113)

^*^97 cases analysed, 41 missing.

^**^101 cases analysed, 37 missing.

^***^For 79 patients put on ART.

Presenting characteristics for 138 children with HIV at Arba Minch Hospital, who initiated pre-ART care during the period 2003–2008.

### Differences between age groups

The distribution of presenting stage for different age groups is shown in Table 2. There were 26 children aged 10–14 years, 6 (23.1%) of these presented in a less advanced stage while the remaining 20 (76.9%) presented in an advanced stage. The higher proportion of children presenting late in the oldest age group was found to be statistically significant when compared to the reference group of 0–4 year olds (Chi-square test, χ^2^ = 8.8, P = 0.01). No significant difference was found between the middle and the youngest age group.

**Table 2:** **Presenting stage of HIV/AIDS for different age groups**

	Less advanced (%)	Advanced (%)	Total	χ^2^ significance
**0-4 years** ^*^	33 (55.0)	27 (45.0)	60	-
**5-9 years**	29 (55.8)	23 (44.2)	52	P > 0.05
**10-14 years**	6 (23.1)	20 (76.9)	26	P = 0.01

^*^reference category.

Less advanced = Stage I & II combined.

Advance = Stage III & IV combined.

Presenting stage of HIV/AIDS for different age groups in a cohort of 138 children who initiated pre-ART care during the period 2003–2008.

Pre-ART loss to follow-up proportion within different age groups is shown in Table 3. Among the 78 children who were 5 years or older, 17 (21.8%) were lost to follow-up. The number of children lost to follow-up among the 60 children aged 0–4 years was 24 (40.0%), a significantly higher proportion compared to the older children. (Chi-square test, χ^2^ = 5.4, P = 0.02).

**Table 3:** **Pre-ART loss to follow-up for different age groups**

	Not lost to follow-up (%)	Lost to follow-up (%)	Total	χ^2^ significance
**0-4 years**	36 (60.0)	24 (40.0)	60	P = 0.02
**5-14 years**	61 (78.2)	17 (21.8)	78	

Loss to follow-up for different age groups in a cohort of 138 children who initiated pre-ART care during the period 2003–2008.

### Risk factors for longer time to ART initiation

The 138 patients enrolled in the study contributed 47.0 person years of observation (PYO) in pre-ART follow-up. The median time to ART initiation for all participants was 18 days (IQR 6–113). When controlling for sex, place of residence, enrolment phase and WHO clinical stage upon presentation, it was found that children in the age group 0–4 years waited longer to initiate ART (HR: 0.50, 95% CI: 0.25-0.97, P = 0.04).

**Figure 2:**
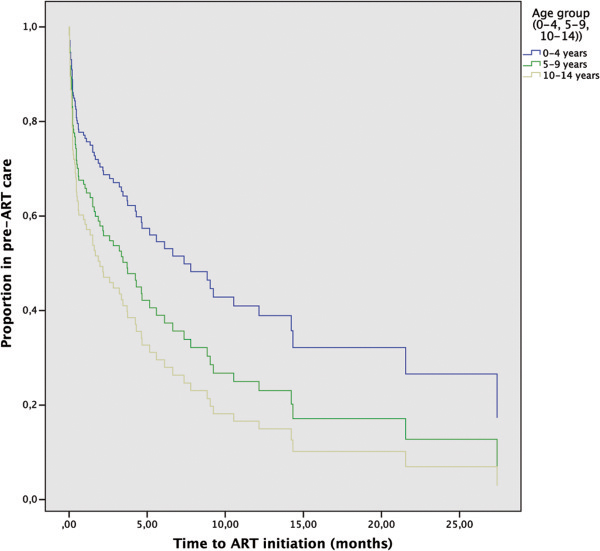
**Time to ART for different age groups.** Survival curves according to Cox regression analysis showing time to ART initiation for different age groups controlling for sex, place of residence, enrolment phase and WHO clinical stage upon presentation. Survival times for patients not reaching the event during the time of their observation (censored data) are also accounted for in the figure.

A longer time to ART initiation was also found for children presenting in stage I (HR: 0.27, 95% CI: 0.09-0.80, P = 0.02). Table 4 shows the adjusted hazard ratios for all variables mentioned above and Figure 2 shows the survival curves according to Cox regression with separate lines for different age groups.

**Table 4:** **Factors associated with longer waiting time to ART initiation, Arba Minch Hospital, Ethiopia**

Variable		Adjusted HR (95% CI)	P-value
**Sex**	Male vs Female	0.90 (0.57-1.42)	>0.05
**Address**	Rural vs Urban	0.98 (0.46-2.05)	>0.05
**Phase of enrolment**	Early phase	1.36 (0.78-2.37)	>0.05
	Rapid scale-up	1.09 (0.59-2.00)	>0.05
	Recent phase^*^	1.00	
**Age group**	0-4 years	0.50 (0.25-0.97)	0.04
	5-9 years	0.40 (0.42-1.41)	>0.05
	10-14 years^*^	1.00	
**WHO clinical stage**	Stage I	0.27 (0.09-0.80)	0.02
	Stage II	0.69 (0.27-1.71)	>0.05
	Stage III	0.88 (0.38-2.04)	>0.05
	Stage IV^*^	1.00	

^*^reference category.

Hazard ratios for factors associated with having a longer time to ART initiation in a cohort of 138 children who initiated pre-ART care during the period 2003–2008.

### Differences between sexes

The proportion of patients presenting in an advanced stage (in either stage III or stage IV) for boys and girls respectively is presented in Table 5. Out of the 59 girls in the sample, 35 (59.3%) presented in an advanced stage; for the boys the number was 35 (44.3%) out of 79. The table also shows sex difference in presenting stage stratified by different age groups. For ages 10–14, all 12 (100.0%) of the girls presented late, as compared to 8 (57.1%) out of the 14 boys and this distribution was determined to be statistically significant (Fisher’s exact test, P = 0,02). However, the sample size was critically small for further statistical evaluation. The sex differences for the other two age groups were not statistically significant, neither was the difference between sexes for all age groups combined.

**Table 5:** **Sex difference in presenting stage of HIV/AIDS**

		Less advanced (%)	Advanced (%)	Total	Significance
**All ages**	**Girls**	24 (40.6)	35 (59.3)	59	
	**Boys**	44 (55.6)	35 (44.3)	79	
**0-4 years**	**Girls**	11 (47.8)	12 (53.2)	23	P > 0.05^a^
	**Boys**	22 (59.5)	15 (40.5)	37	
**5-9 years**	**Girls**	13 (54.2)	11 (45.8)	24	P > 0.05^a^
	**Boys**	16 (57.1)	12 (42.9)	28	
**10-14 years**	**Girls**	0 (0.0)	12 (100.0)	12	P = 0.02^b^
	**Boys**	6 (42.9)	8 (57.1)	14	

^a^Chi-square significance test.

^b^Fisher’s exact test.

Less advanced = Stage I & II combined.

Advanced = Stage III & IV combined.

Sex difference in presenting stage of HIV/AIDS stratified by age group in a cohort of 138 children who initiated pre-ART care during the period 2003–2008.

Neither boys nor girls were found to be overrepresented at the clinic. According to official reports, the proportion of girls in need of ART in SNNPR was 49.7% in 2011. It was not possible to obtain this proportion for the actual period studied (2003–2008) but the proportion was not projected to change for the next five years despite an overall estimated decrease in total numbers [4]. On these grounds it was assumed that the proportion was similar during the period of the study. The proportion of girls in the sample was 43.0% – 6.7 percentage points lower than girls in need of ART in the region – but this difference was not found to be significant for P < 0.05 (two-tailed significance, P = 0.10).

### Changes after the scale-up of ART

WHO clinical stage for children presenting to care at Arba Minch Hospital during the three phases of ART scale-up in Ethiopia is shown in Table 6. In the early phase, 17 (41.5%) out of 41 children presented in a less advanced stage, a proportion which in the recent phase rose to 35 (59.3%) out of 59 children. Due to the small size of the sample, an analysis to assess whether this trend was significant was not performed.

**Table 6:** **Presenting stage during the different phases of ART scale-up**

	Less advanced (%)	Advanced (%)	Total
**Early phase**	17 (41.5)	24 (58.5)	41
**Rapid scale-up phase**	16 (42.1)	22 (57.9)	38
**Recent phase**	35 (59.3)	24 (40.7)	59

Less advanced = Stage I & II combined.

Advanced = Stage III & IV combined.

Presenting stage during the different phases of ART scale-up in a cohort of 138 children who initiated pre-ART care during the period 2003–2008.

## Discussion

This study shows that older children present later to care and that among the older children, girls present later than boys. Younger children face other problems, as they are shown to have a longer waiting time to initiate ART and to be more likely to discontinue their pre-ART program. The overall percentage of pre-ART loss to follow-up is alarmingly high and a notable TB prevalence (14,5%) is seen upon presentation. No sex difference was found in presentation to care among the children in need of ART in the region.

Another study was conducted on HIV-infected adults presenting to care at Arba Minch Hospital during the same period as the children participating in this study. In the adult cohort consisting of 2191 patients, 25% were lost to pre-ART follow-up [6]. As was discussed earlier, the ART coverage of children in Ethiopia is lower than that of adults [22]. This fact together with the loss to follow-up reported for adults at the same hospital suggest a worrying pattern where children not only have less access to care but also continue care to a lesser extent than adults. A likely explanation for this is the fact that the children presenting to care in this study were generally healthier (in a less advanced stage of HIV) than the adults but even so the issue ought not be disregarded. It is however worth noting that the new WHO guidance of starting ART for all children under 5 has been endorsed by the Ethiopian Ministry of Health but its cost and associated implications are being studied before its implementation. If implemented, this new guidance may alleviate some of the concerns noted in this study.

It may not be surprising that older children presented later. Studies from around world show that – although figures vary slightly in different settings – a majority of HIV infected children anywhere have acquired their infection vertically. If this is the case also for the area serviced by Arba Minch Hospital, it would mean the disease had had longer time to progress in the children aged 10–14 years. Yet, if perinatal transmission is indeed the dominating mode among these children, it is still troubling that so many cases go undiscovered so long – one in five being over 9 years old and the better part being 5 years or older. This is not unique for Ethiopia. Age distributions such as these have been reported elsewhere in low-income countries; a recent Ugandan study showed roughly similar proportions at two different district hospitals and children’s ages were also high in a large Indian cohort [23, 24]. The fact that older children present later could also be part of the explanation why younger children are lost to pre-ART follow-up more frequently, as being in a less advanced stage has been shown elsewhere to be a predictor for pre-ART loss to follow-up [6].

As for the older girls presenting later than the older boys, potential explanations are less obvious. Studies on adults have actually reported women in Sub-Saharan Africa to present earlier than men, but additional factors associated with early presentation are pregnancy and having children less than 5 years [6, 15]. These factors are naturally less occurrent among younger girls. Younger girls are also much less likely to be married, and being single is associated with presenting late not only in general but especially for women [15]. This kind of extrapolation however is somewhat speculative and probably does not tell the whole story.

On the other hand, previous research may provide a more straightforward explanation for the high loss to follow-up and longer waiting times among the youngest children. One reason that sufficient adherence can be hard to achieve for children is that they have to rely on their caregivers for it, caregivers who are often themselves in poor health due to HIV or difficult social circumstances [25]. The youngest children being most reliant on adults, this could explain their low retention. It could also explain the longer waiting time, since it is not recommended to initiate ART before it has been properly established that the patient is likely to adhere to the treatment [18, 19].

TB is the leading cause of death for people living with HIV and ART has been shown to substantially reduce the incidence of TB [22, 26]. Therefore it is important to note the high rates of previous and current TB infection among the children presenting to care. Sadly, these findings are not that surprising, seeing that high rates have been reported in other child cohorts from low-income countries [11, 25, 27]. In any event, the findings underline the importance of earlier initiation of ART and generally improving collaborative TB-HIV care.

The main limitation of this study is the small sample size. Due to this fact, changes in presenting stage and mortality after the rapid scale-up of ART could not be analysed properly. This is unfortunate since these and other factors may have improved as a result of the scale-up, as has been shown for the adult cohort. On some instances when analyses were made on sub-groups of the entire cohort the sample size was even smaller. Therefore the finding that older girls present later than boys should be interpreted with caution. The large number of tests performed on this rather small sample also increases the risk of finding false significance and this should be taken into consideration.

Another limitation of this study was that the circumstances of data collection did not allow for a separate set of variables to be recorded for the child cohort. Information on parents’ infections and social status, measures of prevention during pregnancy, mode of delivery and nutritional status of the child would have been valuable supplements for the analyses.

The range of problems seen in the provision of ART and retention in care all point to the same basic conclusion: reducing the rate of mother-to-child transmission is key to improving the paediatric HIV situation in Ethiopia. More research is needed to assess how perinatal care for infected women as well as testing of and prophylaxis for infants can be improved upon.

Nonetheless in addition to this, as long as there are still children infected with HIV, the detection of and care for these children need improvement as well. More research should explore the factors associated with loss to follow-up, late presentation, not presenting to care at all and possible interventions to solve these problems. Although likely candidates have been suggested in this discussion for the most immediate causative factors, there are likely also more general social factors such as poverty that put children at risk for lower access to care, lower adherence and unfavourable disease outcome. For this reason, more social research in addition to purely medical research would be welcome. To determine changes over time in pre-ART and on-ART loss to follow-up, mortality and presenting stage among children, research on larger Ethiopian cohorts of children is of crucial importance in the future.

Some inequalities between patient groups described in this study may still be prevalent despite the general improvement after the ART scale-up. Thus, even though our data are by now a few years old the results are nonetheless relevant in this respect and hopefully our findings will also serve a purpose for comparison with the contemporary adult cohort, as well as with future studies of this kind.

## Conclusions

Although the sample size in this study was too small to make some important analyses on how the situation has developed over time, a number of problems have been identified concerning HIV-infected children presenting to care. The main ones are high pre-ART loss to follow-up rate, high TB prevalence and late presentation. Reasons for the higher loss to follow-up and longer waiting time to initiate treatment among the youngest children need to be further investigated. So do potential reasons for older girls presenting later than older boys and also general social factors that could be associated with several of these problems.

## Electronic supplementary material

Additional file 1:**STROBE 2007 (v4) Statement—Checklist of items that should be included in reports of cohort studies.**(PDF 95 KB)

## Abbreviations

AIDS: Aquired Immunodeficiancy Syndrome
ART: Antiretroviral therapy
ARV: Antiretroviral drug
CI: Confidence interval
HIV: Human immunodeficiancy virus
HR: Hazard ratio
MOH: Ministry of Health (Ethiopia)
TLC: Total lymphocyte count
SNNPR: Southern Nations Nationalities and Peoples’ Region
SPSS: IBM SPSS statistics software
TB: Tuberculosis
WHO: World Health Organisation.
