# Supplementary material for: Pre-ART retention in care and prevalence of tuberculosis among HIV-infected children at a district hospital in southern Ethiopia
Source: BMC Pediatr. 2014 Oct 4;14:250. doi: 10.1186/1471-2431-14-250 (PMC4287195; doi:10.1186/1471-2431-14-250)
Supplement: Supplementary file 1 — Additional file 1:STROBE 2007 (v4) Statement—Checklist of items that should be included in reports of cohort studies.(PDF 95 KB) [file 12887_2014_1219_MOESM1_ESM.pdf]

STROBE 2007 (v4) Statement—Checklist of items that should be included in reports of *cohort studies*

(MS: 1447915263125390)

Pre-ART retention in care and prevalence of tuberculosis among HIV-infected children at a district hospital in southern Ethiopia

Emil Westerlund Degu Jerene Zewdie Mulissa Inger Hallström and Bernt Lindtjørn

19th September 2014

Section/Topic	Item #	Recommendation	Reported on page #
**Title and abstract**	1	(*a*) Indicate the study's design with a commonly used term in the title or the abstract	Page 2
		(*b*) Provide in the abstract an informative and balanced summary of what was done and what was found	Pages 2-3
**Introduction**			
Background/rationale	2	Explain the scientific background and rationale for the investigation being reported	Pages 2-3
Objectives	3	State specific objectives, including any prespecified hypotheses	Page 5
**Methods**			
Study design	4	Present key elements of study design early in the paper	Pages 5-6
Setting	5	Describe the setting, locations, and relevant dates, including periods of recruitment, exposure, follow-up, and data collection	Pages 5-9
Participants	6	(*a*) Give the eligibility criteria, and the sources and methods of selection of participants. Describe methods of follow-up	Pages 5-6, page 8
		(*b*) For matched studies, give matching criteria and number of exposed and unexposed	N/A
Variables	7	Clearly define all outcomes, exposures, predictors, potential confounders, and effect modifiers. Give diagnostic criteria, if applicable	Pages 7-10
Data sources/ measurement	8*	For each variable of interest, give sources of data and details of methods of assessment (measurement). Describe comparability of assessment methods if there is more than one group	Page 7
Bias	9	Describe any efforts to address potential sources of bias	Page 10
Study size	10	Explain how the study size was arrived at	Page 6
Quantitative variables	11	Explain how quantitative variables were handled in the analyses. If applicable, describe which groupings were chosen and why	

Statistical methods	12	(*a*) Describe all statistical methods, including those used to control for confounding	Pages 9-10
		(*b*) Describe any methods used to examine subgroups and interactions	Page 10
		(*c*) Explain how missing data were addressed	N/A
		(*d*) If applicable, explain how loss to follow-up was addressed	Page 10
		(*e*) Describe any sensitivity analyses	N/A
**Results**			
Participants	13*	(a) Report numbers of individuals at each stage of study—eg numbers potentially eligible, examined for eligibility, confirmed eligible, included in the study, completing follow-up, and analysed	Pages 10-11
		(b) Give reasons for non-participation at each stage	Pages 10-11
		(c) Consider use of a flow diagram	Figure 1
Descriptive data	14*	(a) Give characteristics of study participants (eg demographic, clinical, social) and information on exposures and potential confounders	Table 1, pages 14-16
		(b) Indicate number of participants with missing data for each variable of interest	N/A
		(c) Summarise follow-up time (eg, average and total amount)	Page 11-12
Outcome data	15*	Report numbers of outcome events or summary measures over time	Pages 11/Figure 1
Main results	16	(*a*) Give unadjusted estimates and, if applicable, confounder-adjusted estimates and their precision (eg, 95% confidence interval). Make clear which confounders were adjusted for and why they were included	Pages 11-14
		(*b*) Report category boundaries when continuous variables were categorized	Pages 11-12
		(*c*) If relevant, consider translating estimates of relative risk into absolute risk for a meaningful time period	N/A
Other analyses	17	Report other analyses done—eg analyses of subgroups and interactions, and sensitivity analyses	Pages 12-14
**Discussion**			
Key results	18	Summarise key results with reference to study objectives	Pages 17
**Limitations**			
Interpretation	20	Give a cautious overall interpretation of results considering objectives, limitations, multiplicity of analyses, results from similar studies, and other relevant evidence	Pages 14-18
Generalisability	21	Discuss the generalisability (external validity) of the study results	Pages 14-18
**Other information**			
Funding	22	Give the source of funding and the role of the funders for the present study and, if applicable, for the original study on which the present article is based	Pages 18-19

*Give information separately for cases and controls in case-control studies and, if applicable, for exposed and unexposed groups in cohort and cross-sectional studies.

Note: An Explanation and Elaboration article discusses each checklist item and gives methodological background and published examples of transparent reporting. The STROBE checklist is best used in conjunction with this article (freely available on the Web sites of PLoS Medicine at http://www.plosmedicine.org/, Annals of Internal Medicine at http://www.annals.org/, and Epidemiology at http://www.epidem.com/). Information on the STROBE Initiative is available at www.strobe-statement.org.
